# Utilising Quick Response (QR) Codes to Provide Patient Information Leaflets in Skin Cancer Clinics: Educational, Financial and Environmental Benefits

**DOI:** 10.7759/cureus.102982

**Published:** 2026-02-04

**Authors:** Niall O'Hara, Wayne Jaffe

**Affiliations:** 1 Plastic and Reconstructive Surgery, Queen Elizabeth Hospital Birmingham, Birmingham, GBR; 2 Plastic and Reconstructive Surgery, University Hospital North Midlands, Stoke-on-Trent, GBR

**Keywords:** mobile technology, patient education, patient information leaflet, qr code, skin cancer

## Abstract

Skin cancer clinics play a pivotal role in early detection and effective management of skin cancer. Patient education is a key component in achieving these goals. This observational evaluation study explores our experience of the introduction of quick response (QR) code technology to access patient information leaflets (PILs) in skin cancer clinics. We discuss the evidence behind and previous use of QR code technology in clinical settings and highlight the potential educational, cost-saving and environmental benefits. By harnessing the power of QR codes, clinicians can enhance patient education, facilitate informed decision-making, and optimise healthcare resource utilisation.

## Editorial

Skin cancer is a common and potentially life-threatening condition that requires early detection and appropriate care with shared decision-making between the patient and clinician, potentially for several years. It is also becoming more common with time. The incidence of melanoma rose by 44% between 2008 and 2018, and it is predicted that by 2040, this will rise by a further 62% [1]. However, its mortality-to-incidence ratio shows that with early detection and appropriate management, melanoma remains one of the most treatable cancers. Patient education is therefore vital for raising awareness, promoting self-care practices, and improving health outcomes.

In dermatology and plastic surgery skin cancer clinics, there is a wealth of information a clinician needs to pass on to the patient: their diagnosis, the treatment options available and their associated risks, strategies to reduce the risk of developing future skin lesions, skin and lymph node self-examination, and the potential role of sentinel lymph node biopsy. Patients can come away from the clinic feeling overwhelmed with information. Studies show that 40% to 80% of medical information provided by healthcare practitioners is forgotten immediately. The greater the amount of information presented, the lower the proportion correctly recalled. Furthermore, almost half of the information that is remembered is incorrect [2]. In skin cancer clinics, the use of sentinel lymph node biopsy as a potentially diagnostic but not necessarily therapeutic surgical option can be an especially tricky aspect to explain, all the more so in a consultation where a patient may already be anxious after receiving a new cancer diagnosis.

Patient information leaflets (PILs) are widely used by clinicians to reinforce and supplement information discussed during a consultation. Numerous studies have demonstrated the positive impact of information leaflets on patient education. A combination of written and verbal information has been shown to be more effective in improving patient knowledge and satisfaction compared to verbal information alone. For cancer patients, information leaflets also diminish levels of anxiety [3].

The British Association of Dermatologists (BAD) has written comprehensive PILs on a wide range of dermatological conditions, including skin cancers and pre-cancerous conditions. These leaflets offer evidence-based information on skin cancers, risk factors, symptoms, preventive measures and early detection techniques, as well as clear explanations of treatment options and rationales. They empower patients with knowledge and encourage self-examination, leading to earlier detection of future suspicious lesions. Moreover, PILs facilitate patient-clinician communication, enabling informed discussions and shared decision-making.

Despite a global push for sustainable practices and the almost ubiquitous ownership of mobile smartphones, PILs are still mostly disseminated in printed form. In recent years, BAD has created quick response (QR) codes, which enable quick and easy access to each of their leaflets by scanning the code with a smartphone. We have implemented the use of these QR codes in all skin cancer consultations where a relevant BAD leaflet is available. Nursing staff in our clinic created a physical folder with PILs listed in alphabetical order and a large QR code on every single page. We have adopted the practice of inviting patients in the skin cancer clinic to scan the QR code(s) relevant to their consultation, with the option in reserve of printing leaflets where appropriate, for example, for those patients who do not have access to a smartphone. We have also adopted the practice of including the QR code and the relevant BAD website link in the hospital letter sent to patients in the post after their clinic consultation.

There have been some previous similar practices undertaken in sexual health clinics, fracture clinics, orthodontic clinics and eye clinics, which have all demonstrated improvements in patient satisfaction [4,5]. All of these studies demonstrated that patients found QR codes easier to access and preferable to paper copies of information leaflets. Initial feedback from our own patients has been exceedingly positive, and clinicians have found that easy access to the PILs in QR code form has increased patient engagement and ownership of their health condition and saves time in the clinic rather than having to retrieve or print out a physical copy of the leaflet.

We have found that the use of a QR code to access an online version of PILs rather than a physical paper copy offers a multitude of logistical benefits. The online leaflets have hyperlinks which can take the user to further explanations in another leaflet (for example, when imiquimod cream is mentioned in the basal cell carcinoma leaflet). In the online leaflets, links, videos and email addresses will open automatically when clicked. Online leaflets allow patients with visual impairment or difficulties to view the content in any font size or brightness they require. QR codes of the BAD information leaflets can also direct patients to multilingual resources, accommodating diverse patient populations. Online versions of leaflets can be bookmarked or downloaded for ease of access at any time, compared to a paper copy, which is easily lost, along with any potential patient education benefits.

In light of the recent COVID-19 pandemic, there is also a reduced risk of cross-contamination with QR code use. In situations where physical leaflets may pose a risk of contamination, such as during infectious disease outbreaks, QR codes provide a contactless alternative.

We have found several benefits to patient education through our use of QR code PILs. We have found that when our patients return for surgery or follow-up, they have an increased understanding of their own condition, the potential risks and side effects of the management options they are being offered and the follow-up care that they can expect. We have found that the QR code leaflets have facilitated shared decision-making in the clinic. Our QR code PILs have empowered our patients to engage in informed discussions with their medical team, actively participate in their care decisions and select the most suitable treatment plan for themselves as an individual.

There are also several cost-saving benefits to the use of a QR code over that of a traditional paper leaflet. After the initial cost of the creation of our QR code portfolio folder, we have rarely had to print a further physical leaflet. Moreover, with physical leaflets, updating information or adding additional resources can be challenging and costly. In contrast, QR-accessed online leaflets allow for easy, regular updates and expansions as guidelines change and patients give their feedback. With improvements to early detection and self-examination, QR code PILs contribute to less advanced stages of skin cancer, reducing the need for extensive surgical procedures, expensive therapies and prolonged hospital stays. We have also experienced a reduced need for follow-up consultations or phone calls, reducing the burden on clinicians.

In October 2020, the NHS became the world’s first health service to commit to reaching carbon net zero. The Greener NHS programme aims to save money and reach net carbon zero [6]. QR codes eliminate the need for printing, storing and distributing physical leaflets. This can be particularly beneficial for clinics with limited resources or those aiming to reduce paper waste and environmental impact.

We did find some limitations to the use of QR-access PILs. The patient population typically presenting with skin cancers are older and may not always have access to a smartphone with a suitable camera to scan QR codes. Patchy access to hospital Wi-Fi can slow or limit access to online information. However, we found that these difficulties were overcome relatively easily and that the vast majority of patients preferred the online version compared to traditional PILs.

The use of QR-accessed PILs also opens the avenue to exciting future applications. Online leaflets can provide easy access to additional dynamic multimedia resources such as videos, interactive animations, and quizzes, enhancing patient education, knowledge retention and engagement. Videos could include patient testimonials or step-by-step instructional videos on performing accurate self-examinations for new skin lesions, correct application of sunscreen, appropriate clothing choices and shade-seeking behaviour. QR codes could also be designed to track usage and gather valuable analytics data so that clinicians could gain insights into patient engagement and preferences. These data could inform future improvements in educational materials and help tailor information to better meet patient needs. We plan to analyse patient outcomes after our QR-accessed PILs are in place for some time to quantifiably measure the cost-benefit, patient satisfaction, and improved patient knowledge, and to expand the QR codes available to include locally produced hospital-specific leaflets.

Patient education through information leaflets plays a crucial role in skin cancer clinics, promoting early detection and empowering patients to make informed decisions about their care. By incorporating QR code technology, clinics can enhance patient education, improve engagement and optimise healthcare resource utilisation, resulting in significant cost-saving benefits for patients and the healthcare system as a whole. QR codes offer a modern, efficient and flexible approach to patient education. By leveraging this technology, clinics can improve accessibility, interactivity and cost-effectiveness while adapting to sustainable environmental practices and tailoring information to individual patient needs.
